# Comparison of Transvaginal and Transperineal Ultrasonographic Uterocervical Angle Measurements in Low-Risk Pregnancies at 24–34 Weeks’ Gestation

**DOI:** 10.3390/diagnostics15243232

**Published:** 2025-12-17

**Authors:** Emrah Dagdeviren, Yucel Kaya

**Affiliations:** 1 Department of Obstetrics and Gynecology, Basaksehir Cam and Sakura City Hospital, Istanbul 34480, Turkey; 2 Division of Perinatology, Department of Obstetrics and Gynecology, Basaksehir Cam and Sakura City Hospital, Istanbul 34480, Turkey; yucelkaya0007@gmail.com

**Keywords:** agreement, consistency, correlation, preterm birth, transperineal ultrasound, transvaginal ultrasound, uterocervical angle

## Abstract

**Background:** The uterocervical angle (UCA) is a promising ultrasound parameter for predicting preterm birth. Transvaginal ultrasound (TVUS) is the gold standard for cervical assessment; however, some patients may decline the procedure due to discomfort or embarrassment. Although transperineal ultrasound (TPUS) offers an alternative associated with less discomfort, comparative data on UCA measurements between these two methods are limited. **Objective:** We aimed to evaluate the consistency and agreement between UCA measurements obtained using TVUS and TPUS in pregnant women between 24 and 34 weeks of gestation. **Methods:** In this prospective cross-sectional study, UCA and CL measurements of 189 pregnant women between 24 and 34 weeks of gestation were performed using TVUS and TPUS by a single specialist. Of these, 25 women (13.2%) were excluded due to inadequate TPUS image quality. A total of 164 women were included in the statistical analysis. Pearson correlation analysis, intraclass correlation coefficient (ICC), Lin’s concordance correlation coefficient (CCC), and Bland–Altman analysis were performed. **Results:** UCA measurements showed a high positive correlation between TVUS and TPUS (*r* = 0.833, *p* < 0.001). The ICC was 0.827 (95% CI: 0.77–0.87), indicating good consistency, and the CCC was 0.81 (95% CI: 0.76–0.86). The Bland–Altman analysis demonstrated a median difference of 3° between UCA measurements obtained via TVUS and TPUS. The non-parametric limits of agreement, represented by the 2.5th and 97.5th percentiles, ranged from −20.9° to 34.8°. **Conclusions:** TPUS shows insufficient agreement to be used interchangeably with TVUS for UCA measurement. Although the level of consistency is high, inadequate image quality in a subset of cases and the uncertainty regarding the clinical utility of TPUS-derived measurements for predicting preterm birth limit its current clinical applicability.

## 1. Introduction

Preterm birth remains one of the leading causes of perinatal morbidity and mortality worldwide [1]. Early identification of women at risk and timely diagnosis are essential for effective intervention. Preterm labor is defined as regular uterine contractions leading to cervical changes before 37 weeks of gestation [2] and clinically it is typically confirmed by regular uterine contractions (six or more per hour) accompanied by either cervical dilatation of ≥3 cm [3], a cervical length (CL) < 20 mm on transvaginal ultrasound (TVUS) [4,5,6], or a CL between 20–29 mm with a positive fetal fibronectin test [4,5,6].

In recent years, numerous studies have been conducted on the anatomical angulations of the uterus beyond CL [7,8,9,10,11]. The uterocervical angle (UCA), a novel sonographic parameter, has recently emerged as a potential marker in predicting preterm birth [7,12,13]. A wide UCA may create a more direct pathway between the uterus and the cervix, potentially increasing the pressure exerted by the fetus and its appendages on the cervix. This mechanism is thought to potentially contribute to the opening of the cervical barrier during preterm birth. Moreover, combined assessment of CL and UCA between 24 and 34 weeks in women with threatened preterm labor has been reported to offer improved predictive accuracy for spontaneous preterm birth [14]. Although research on UCA is ongoing, it is expected to become a valuable tool in preterm birth prediction. In a study, it has been reported that, beyond preterm birth, the combination of UCA with the Bishop score may be effective in predicting successful labor induction [11]. Considering clinical situations such as an unfavorable cervix increasing the risk of cesarean delivery [15], the UCA emerges as a parameter that may have potential clinical utility in various obstetric scenarios.

While TVUS remains the gold standard for assessing CL [6], it involves inserting a probe into the vagina, which some patients may decline due to discomfort or embarrassment [16]. Transperineal ultrasound (TPUS), performed by placing the probe externally over the labia, offers a less discomforting alternative. In clinical scenarios where TVUS is contraindicated, such as in cases of placenta previa or suspected preterm premature rupture of membranes, TPUS may be particularly advantageous [17,18]. After the 24th gestational week, CL measurements obtained via TPUS have demonstrated both agreement [19] and correlation [20] with those obtained via TVUS. A recent study reported a statistically significant correlation but weak consistency between TPUS and TVUS for UCA measurements in pregnancies between 16 and 24 weeks of gestation [21]. The comparison of UCA measurements between TVUS and TPUS has not been performed in pregnancies between 24 and 34 weeks of gestation.

### Objectives

Our primary aim is to evaluate the consistency and agreement between UCA measurements obtained using TPUS and TVUS in pregnant women between 24 and 34 weeks of gestation. Our secondary aim is to evaluate the correlation and the difference in UCA and CL values between the two methods.

## 2. Materials and Methods

### 2.1. Study Design

This was a prospective cross-sectional study conducted between November 2024 and October 2025. The study was conducted at a tertiary care center. Approval for the study was obtained from the hospital’s local ethics committee (KAEK-11/16.10.2024.187, approved on 23 October 2024), and written informed consent was obtained from all participants prior to enrollment.

### 2.2. Participants

Inclusion criteria were as follows: pregnant women aged 18–40 years, gestational age between 24 + 0 and 33 + 6 weeks, singleton pregnancy, vertex presentation, absence of uterine contractions, and a Bishop score less than 6. Exclusion criteria included: history of preterm birth, use of progesterone for preterm birth prevention, fetal anomalies, placenta previa, chronic maternal diseases (e.g., hypertension, diabetes), non-vertex presentation, multiple gestations, history of previous cesarean section and uterine surgery, abnormal Pap smear, presence of cervical cerclage, history of loop electrosurgical excision procedure (LEEP) or conization, cervical surgery, or dilation and curettage, as well as patients with acoustic shadowing caused by the symphysis pubis and rectum during TPUS examination.

### 2.3. Data Sources/Measurement

All TVUS and TPUS measurements were performed by a single experienced specialist following bladder emptying, and in the absence of uterine contractions, to minimize distortion of pelvic anatomy. To account for the cervix’s dynamic structure, three separate measurements were obtained per patient, and UCA and CL were measured from three different ultrasound images. In accordance with current literature recommendations, the shortest valid CL measurement was used for analysis [22,23]. For UCA, the value from the same ultrasound image corresponding to the shortest CL measurement was used for analysis. Ultrasound assessments were conducted using the Hitachi Arietta 65 Ultrasound system (Tokyo, Japan). TVUS was performed using a 4–9 MHz endocavitary vaginal probe, and TPUS using a 1–5 MHz C253 convex abdominal probe.

**Figure 1:**
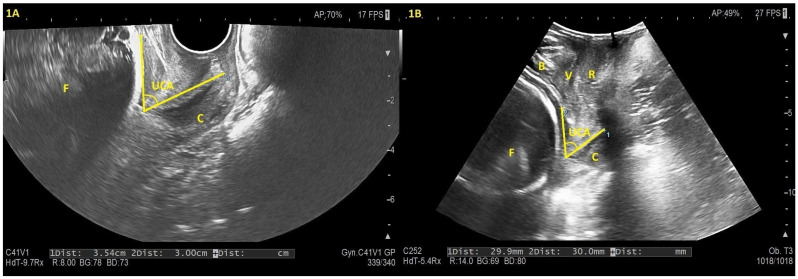
Transvaginal (**1A**) and transperineal (**1B**) ultrasound images of a 30-week pregnancy. In both images, the uterocervical angle (UCA) is indicated by the dotted lines and curved bracket. The fetus (F) is visible in both sections. In the transperineal image (**1B**), the bladder (B), vagina (V), and rectum (R) are also labeled for anatomical reference. Abbreviations: B: Bladder; C: Cervix; F: Fetus; R: Rectum; UCA: Uterocervical angle; V: Vagina.

CL was measured in the dorsal lithotomy position, defined as the distance between the internal and external cervical os, following the International Society of Ultrasound in Obstetrics and Gynecology (ISUOG) guidelines [23]. In cases of cervical curvature, the total CL was obtained by summing measurements across two or three linear segments [24]. UCA was measured according to the method described by Dziadosz et al. [13]. The first line of the angle was drawn along the endocervical canal, defined as the straight line from the internal to the external os, regardless of curvature. The second line extended from the internal os along the anterior uterine segment for 3 cm. The angle between these two lines was recorded as the UCA in Figure 1. In the presence of funneling, the UCA was measured as described by Dziasdzos et al. [14]. The first ray was placed to measure the length of the remaining cervix, and the second caliper was positioned from the innermost portion of measurable cervix and extended to the lower uterine segment. If the lower uterine segment was irregular, the second caliper was placed centrally along the segment.

TPUS measurements of CL and UCA were performed in the dorsal lithotomy position after bladder emptying in Figure 1. The points and axes defined for UCA and CL measurement with TVUS were similarly applied for TPUS measurements. For hygiene purposes, the TPUS convex probe was covered with a glove and placed on the perineum along the midsagittal line [25]. We followed a standardized protocol consistent with current clinical practice and previously published TPUS methodology. Specifically, the operator used a minimal and constant probe pressure, kept the probe in a mid-sagittal position without exerting additional compression, and applied gentle labial separation only when necessary to optimize the acoustic window. These steps were performed uniformly for all participants. Measurements were obtained after visualizing the bladder, uterus, cervix, vagina, and rectum on the sagittal image, following the methodology outlined by Dietz et al. [26].

### 2.4. Statistical Methods

Statistical analyses were conducted using SPSS software (Version 26.0.1; SPSS Inc., Chicago, IL, USA). Kolmogorov–Smirnov test and histogram were used to assess normality of distribution. Normally distributed data were expressed as mean ± standard deviation and analyzed using the paired *t*-test. Non-normally distributed data were expressed as median (minimum–maximum) and compared using the Wilcoxon signed-rank test. Categorical variables were presented as frequencies (*n*, %) and analyzed using the chi-square test. Correlations between normally distributed variables were evaluated using Pearson correlation analysis [27]. To assess the measurement consistency between the methods, intraclass correlation coefficient (ICC) were calculated using reliability analysis [28]. The ICC model selected was two-way mixed, consistency, single measures, which corresponds to the ICC (3,1) model. To assess the measurement agreement between the methods, Lin’s concordance correlation coefficient (CCC) was calculated. We based the interpretation of the strength of the CCC on the proposal by McBride (2005), a CCC value of less than 0.90 indicates poor agreement, a value between 0.90 and 0.95 indicates moderate agreement, a value between 0.95 and 0.99 indicates substantial agreement, and a value greater than 0.99 indicates almost perfect agreement [29]. Additionally, agreement between measurement methods was evaluated using non-parametric Bland–Altman plots due to the non-normal distribution of differences [30]; these plots illustrated the systematic bias (median difference) and the 2.5th and 97.5th percentiles as non-parametric limits of agreement. A *p*-value of < 0.05 was considered statistically significant. Sample size was calculated using G*Power 3.0 software. For the a priori power analysis, the expected correlation coefficient (*r* = 0.686) between TPUS and TVUS measurements, derived from our previous study, was used as input in G*Power (Correlation: Bivariate normal model, α = 0.05, power = 0.80) [21].

## 3. Results

**Figure 2:**
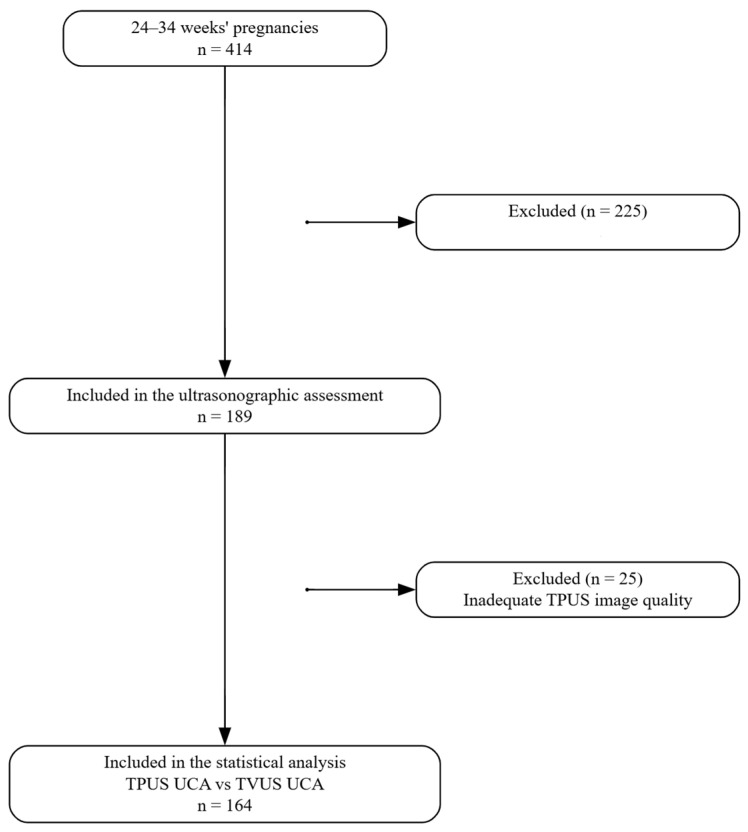
Flowchart of the study. Abbreviations: TPUS, transperineal ultrasound; TVUS, transvaginal ultrasound; UCA, uterocervical angle.

The study flow chart is presented in Figure 2. A total of 189 pregnant women were included in the ultrasonographic evaluation. 25 patients were excluded from the study due to acoustic shadowing from the symphysis pubis and rectum during TPUS application. Statistical analyses were performed with the remaining 164 patients.

### 3.1. Descriptive Data

Demographic characteristics are summarized in Table 1. The median maternal age was 27 years (range: 18–40). The mean body mass index (BMI) was 28.91 ± 5.08 kg/m^2^. The median gestational age at the time of ultrasonographic evaluation was 208 days (range: 168–238). Of the participants, 83 (50.60%) were nulliparity, 36 (21.95%) had a history of previous cesarean section, and 68 (41.46%) were primigravidae. The majority of participants 144 (87.80%) were of Turkish ethnicity.

### 3.2. Outcome Data

Ultrasonographic measurements of CL and UCA obtained via TVUS and TPUS are presented in Table 2. The mean CL measured via TVUS was significantly greater than that measured by TPUS (33.03 ± 5.51 mm vs. 31.78 ± 5.07 mm, *p* < 0.001). In contrast, the mean UCA was significantly wider when assessed by TPUS compared to TVUS (107.16 ± 20.71° vs. 103.85 ± 23.32°, *p* = 0.001).

**Figure 3:**
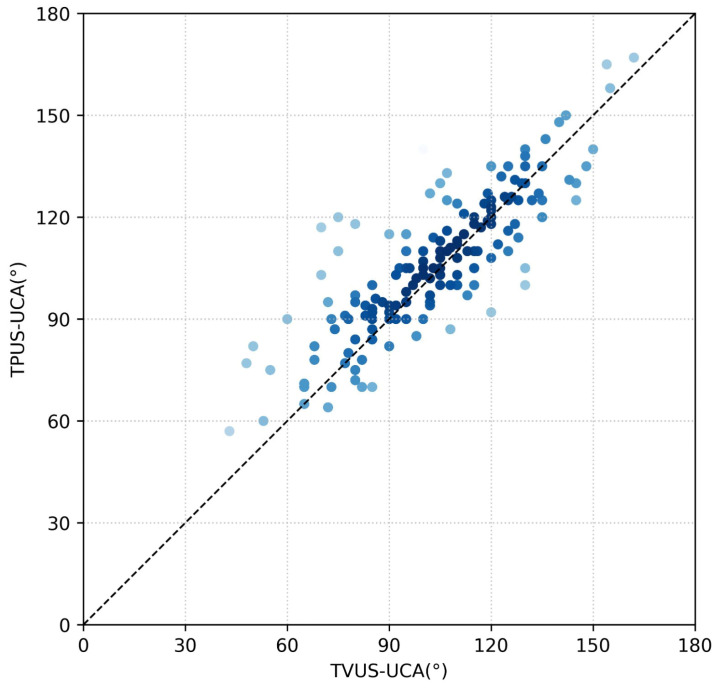
Lin’s concordance correlation between the TVUS and TPUS uterocervical angle measurements was 0.81 (pairs of successful measurements = 164; 95% CI: 0.76–0.86). Abbreviations: TPUS, transperineal ultrasonography; TVUS, transvaginal ultrasonography; UCA, uterocervical angle.

Pearson correlation analysis revealed a high positive correlation between UCA measurements obtained via TVUS and TPUS, with a correlation coefficient of 0.833 (*p* < 0.001), indicating a statistically significant linear relationship. The ICC for UCA measurements between TVUS and TPUS was 0.827 (95% CI: 0.77 to 0.87), indicating good consistency. The CCC between UCA measurements obtained by TVUS and TPUS was 0.81 (95% CI: 0.76 to 0.86) in Figure 3.

**Figure 4:**
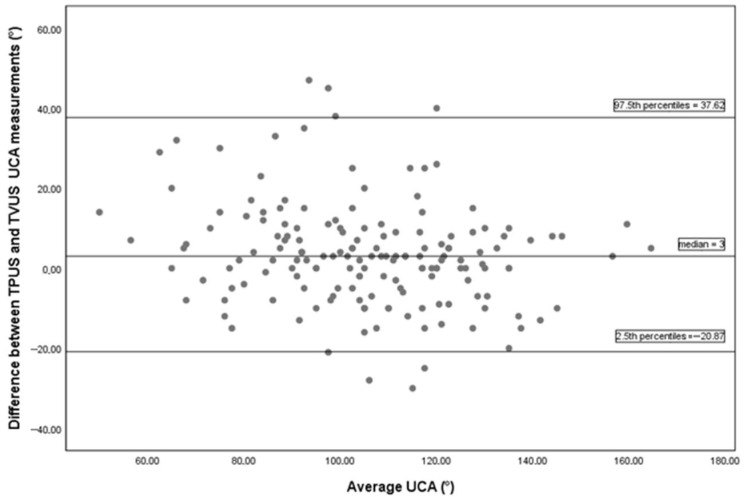
The Bland–Altman plot between TVUS and TPUS uterocervical angle measurements includes 164 pairs of successful measurements. The median difference of 3 degrees between the methods indicates a systematic bias. The 2.5th and 97.5th percentiles of the differences were −20.87 and 37.62 degrees, respectively, representing the non-parametric limits of agreement. Abbreviations: TPUS, transperineal ultrasonography; TVUS, transvaginal ultrasonography.

The Bland–Altman analysis demonstrated a median difference of 3 degrees between UCA measurements obtained via TVUS and TPUS. The non-parametric limits of agreement, represented by the 2.5th and 97.5th percentiles, ranged from −20.87 to 37.62 degrees in Figure 4.

## 4. Discussion

### 4.1. Principle Findings

A high positive correlation was observed between UCA measurements obtained by TVUS and TPUS (Pearson *r* = 0.833, *p* < 0.001). ICC = 0.827 (95% CI: 0.772–0.870) indicated good consistency between the two methods. The CCC value of 0.81 (95% CI: 0.76–0.86), together with the wide limits of agreement observed in the Bland–Altman analysis (−20.87 to 37.62 degrees), indicates weak concordance for exact one-to-one agreement between the measurements.

### 4.2. Interpretation

To our knowledge, two studies have directly compared UCA measurements obtained by different ultrasonographic approaches. In the cross-sectional study conducted by Wongkanha et al., UCA measurements obtained via TAUS and transvaginal TVUS were compared in a population of 256 pregnant women between 16 and 24 weeks of gestation [31]. Wongkanha reported a moderate correlation between UCA measurements obtained by TAUS and TVUS after bladder emptying (*r* = 0.601). In our previous study, we evaluated the correlation, consistency, and agreement of UCA measurements obtained via TAUS, TPUS, and TVUS in pregnancies between 16 and 24 weeks of gestation [21]. In our previous study, we found a statistically significant positive correlation and moderate consistency between TAUS and TVUS (*r* = 0.547, ICC = 0.545), as well as a statistically significant positive correlation but poor consistency between TPUS and TVUS (*r* = 0.686, ICC = 0.052) [21]. In the present study, we evaluated UCA measurements obtained via TVUS and TPUS in pregnancies between 24 and 34 weeks of gestation. In the present study, we found a high positive correlation (*r* = 0.833), indicating good consistency (ICC = 0.827) but weak concordance for exact one-to-one agreement between measurements (CCC = 0.81). The present study was conducted in pregnant women between 24 and 34 weeks of gestation, whereas our previous study included women between 16 and 24 weeks. The main difference between the studies is that they were conducted at different gestational ages. The larger and more developed cervix in more advanced gestational weeks may allow clearer visualization with ultrasonography, which could have resulted in higher correlation and agreement values.

The high correlation observed in our study is insufficient on its own for clinical interpretation. Correlation reflects the strength and direction of the relationship between two measurements; however, it does not indicate whether the two methods can be used interchangeably. In our study, we found weak concordance for exact agreement between the two methods, with a CCC of 0.81. According to McBride (2005), CCC values below 0.90 indicate poor concordance [29]. Additionally, the Bland–Altman analysis demonstrated wide limits of agreement (ranging from −20.87 to 37.62 degrees), further supporting the presence of weak one-to-one agreement between the measurements. Consistency refers to how similarly two measurement methods behave in terms of ranking or relative position. Even if one method consistently yields higher values than the other, the relative differences between measurements may remain preserved; in such cases, consistency can still be high. Agreement, on the other hand, assesses whether two methods can be used interchangeably by evaluating how closely the measurements match. Agreement is more stringent than consistency and is clinically more meaningful. We found good consistency between the two methods with an ICC of 0.827. Although exact agreement was not achieved, this level of consistency may still provide useful guidance for clinical application. However, 25 women (13.2%) were excluded due to inadequate TPUS image quality, which represents an important limitation for the generalizability of the findings. Additionally, in the present study, although the two methods yielded statistically different mean values for UCA and CL, mean differences alone do not provide information about the interchangeability or clinical utility of the methods in agreement studies.

UCA is a newly investigated and promising parameter, and studies involving transperineal ultrasound have been conducted mainly for the assessment of CL. Although the CL literature does not directly support UCA-specific interpretations, it provides a methodological framework regarding the potential effects of transperineal imaging on cervical measurements in general. Dimassi et al. (24–37 weeks of gestation) and Tsakridis et al. (31–34 weeks of gestation) reported strong correlations between TPUS and TVUS in the assessment of CL, with high correlation coefficients of *r* = 0.95 and *r* = 0.96, respectively [20,32]. In addition, Gauthier et al. evaluated the agreement between CL measurements obtained by TPUS and TVUS in women hospitalized between 25 and 34 weeks of gestation due to threatened preterm labor, and the Bland–Altman plot showed that the mean difference was negligible (1.5 mm) [19].

From a technical standpoint, TPUS avoids direct cervical compression, which may be advantageous in preserving the natural curvature and anatomical configuration of the cervix. On the other hand, TPUS is more susceptible to acoustic shadowing and interference from surrounding pelvic structures. In contrast, TVUS allows for higher resolution and proximity to the cervix but may artificially alter the anatomy due to probe-induced pressure. As emphasized by Oliver et al. in the ACR Appropriateness Criteria, TPUS is useful when TVUS is not feasible due to patient discomfort, high risk (e.g., placenta previa, preterm premature rupture of membranes [18]. Image acquisition may be technically more demanding, particularly in early gestation or in the presence of shadowing artifacts; however, appropriate positioning, use of magnification, and operator training can mitigate these challenges.

### 4.3. Strengths and Limitation

Our present study has several strengths. This is the first study to comparatively assess the UCA using both TVUS and TPUS between 24 and 34 weeks of gestation, providing an original contribution to the literature. The measurements were performed under standardized conditions as recommended in the literature, and the data were analyzed using comprehensive statistical methods. However, our present study also has some limitations. The quality of TPUS images may be limited by shadowing from anatomical structures such as the symphysis pubis, rectum, and fetal head, and measurements can vary depending on the operator’s experience. The potential impact of glove use during TPUS on image quality and measurement boundaries cannot be entirely ruled out. Although UCA has been associated with preterm birth, this study did not include follow-up data on birth outcomes. Because the patients included in our study consisted of low-risk pregnancies and most participants were of Turkish origin, the generalizability of the results is limited. All measurements were performed by a single obstetrician, so interobserver variability could not be assessed, which may limit the generalizability of the findings. Ultrasonographic measurements were performed three times; however, using only a single value for analysis limited the assessment of intraobserver variability. Additionally, having the same operator aware of previous results while conducting measurements may have introduced bias. In the future, repeating the measurements under blinded conditions by the same operator would allow assessment of intraobserver variability; this approach could help control potential bias. Because no external studies comparing UCA measurements by TPUS and TVUS are available, partial reliance on our previous work was unavoidable, which carries a limited risk of interpretive bias.

## 5. Conclusions

TPUS demonstrates good consistency but poor agreement with TVUS in measuring the UCA between 24 and 34 weeks of gestation. Because it showed poor agreement, the use of TPUS as a substitute for TVUS does not appear appropriate. Despite demonstrating high consistency, UCA measurement was inadequate in 10.9% of cases, and the clinical utility of TPUS-derived measurements in predicting preterm birth remains unknown. These limitations restrict the generalizability of the findings and their clinical implications. Larger prospective studies are needed to validate these findings and clarify the role of TPUS in routine clinical practice.

## Figures and Tables

**Table 1:** Maternal Demographic Characteristics of the Study Population.

Demographic Data	*n* = 164
Maternal age ^b^ (year)	27 (18–40)
BMI ^a^ (kg/m^2^)	28.91 ± 5.08
Gestational age ^b^ (days)	208 (168–238)
Nulliparity ^c^	83 (50.60)
Previous cesarean section ^c^ *n* (%)	36 (21.95)
Primigravidas ^c^ *n* (%)	68 (41.46)
Turkish ethnicity ^c^ *n* (%)	144 (87.80)

Abbreviations: BMI, body mass index. ^a^ Normal distribution, Mean ± SD ^b^ Non normal distribution, Median (Minimum–Maximum) ^c^ Categorical data, Number (Percentage%).

**Table 2:** Comparison of Cervical Length and Uterocervical Angle Measurements Between TVUS and TPUS.

	TVUS	TPUS	*p*-Value
CL ^a^ (mm)	33.03 ± 5.51	31.78 ± 5.07	<0.001
UCA ^a^ (°)	103.85 ± 23.32	107.16 ± 20.71	0.001

Abbreviations: CL, cervical length; TPUS, transperineal ultrasound; TVUS, transvaginal ultrasound; UCA, uterocervical angle. ^a^ Normal distribution, Mean ± SD.

## Data Availability

The data presented in this study are available on request from the corresponding author due to privacy and ethical restrictions.

## Abbreviations

ACR	American College of Radiology
BMI	Body mass index
CCC	Concordance correlation coefficient
CI	Confidence interval
CL	Cervical length
ICC	Intraclass correlation coefficient
ISUOG	International Society of Ultrasound in Obstetrics and Gynecology
LEEP	Loop electrosurgical excision procedure
TAUS	Transabdominal ultrasound
TPUS	Transperineal ultrasound
TVUS	Transvaginal ultrasound
UCA	Uterocervical angle
